# Harmful and Beneficial Role of ROS 2020

**DOI:** 10.1155/2022/9873652

**Published:** 2022-07-22

**Authors:** Sergio Di Meo, Paola Venditti, Victor M. Victor, Gaetana Napolitano

**Affiliations:** ^1^University of Naples Federico II, Naples, Italy; ^2^University of Valencia, Valencia, Spain; ^3^University of Naples Parthenope, Naples, Italy

Reactive oxygen species (ROS) are molecules deriving from the incomplete reduction of molecular oxygen in the cell. They can be free radicals, atoms, and molecules with an unpaired electron in their outer shell, and because of their chemical structure, ROS are unstable and highly reactive species. Therefore, they are generally short-lived and often leave the subcellular production site after undergoing a reduction process [1]. Thus, ROS may cause oxidative damage to macromolecules such as DNA, proteins, and lipids [2]. More recent findings highlighted that ROS also have important functions in cellular signalling as participants and modifiers of signalling pathways [3]. Although major ROS-sensitive signal transduction pathways have been shown, the research in this field is very active. In our special issue, several research articles identify new involvement of ROS in signalling pathways for triggering pathologies.

A. Nemmar et al. [4] performed *in vivo* experiments to assess the effects of adenine- (0.2% *w*/*w* in feed for 4 weeks) induced chronic kidney disease (CKD) on heart histology, inflammation, oxidative stress, nuclear factor erythroid 2-related factor 2 (Nrf2) expression, and DNA damage. They showed that adenine intake increases the levels of markers of lipid peroxidation measured by malondialdehyde production and 8-isoprostane and the activities of the antioxidant enzymes superoxide dismutase and catalase. Moreover, the immunohistochemical analysis of the hearts also showed an increase in the expression of Nrf2 in cardiomyocytes. The authors concluded that the administration of adenine in mice induces CKD which is associated with cardiac inflammation, oxidative stress, Nrf2 expression, and DNA damage. These results are consistent with another study that reports that pretreatment with nicorandil (K_ATP_ channel opener) causes upregulation of Nrf2 mRNA in the aortic tissues of rats fed with an adenine-rich diet, suggesting that the improvement of oxidative stress in the aortic tissue could reduce the CDK-associated aortic calcifications [5].

Z. Zhao et al. [6] demonstrated that in *in vitro* experiments, the involvement of ROS in the differentiation of cancer stem-like sphere cells enriched the Hep G2 human hepatocellular carcinoma cell line into endothelial cells forming functional blood vessels. In particular, H_2_O_2_ activates the Akt/IKK signalling pathway thus inducing the differentiation process of the tumour blood vessel. This study indicates a possible mechanism of resistance to antiangiogenic agents. The differentiation of cancer stem cells into tumour endothelial cells and, ultimately, into tumour angiogenesis involves the interaction among ROS, the inactivation of the pentose phosphate metabolic pathway, and the activation of autophagy [7]. Knowledge of the origin of tumour blood vessel helps design powerful cancer therapies and suggests that the use of conventional radiotherapy and chemotherapy-based therapies should be reconsidered [7].

Y. Zhao et al. [8] evaluated oxidative stress, mitochondrial function, osteogenic function, and bone formation in *in vivo* and *in vitro* experiments using lipopolysaccharide- (LPS-) induced inflammation models. *In vivo* experiments were performed using 10-week-old C57BL/6J mice characterized by an alveolar bone defect. They were treated with LPS in the absence and presence of cyclosporine A (CsA) for three weeks. *In vitro* experiments were performed using LPS-treated murine osteoblasts in the presence of CsA or an inhibitor of extracellular signal-regulated kinase 1/2 (ERK1/2). *In vivo* results showed that LPS inhibits bone remodelling and promotes the accumulation of oxidative stress in alveolar bone defects. These biochemical changes are reduced by CsA treatment. *In vitro* experiments identified mitochondria as responsible for increased ROS production and oxidative stress in LPS-treated osteoblasts. Oxidative stress reduced the expression of osteogenic differentiation genes by activating the ROS/ERK signalling pathway. Treatment with CsA improved bone remodelling by alleviating oxidative stress caused by LPS. This finding was associated with inhibition of the ERK signalling pathway, suggesting that inflammatory bone diseases can be treated by preserving mitochondrial function and reducing ROS production. In line with these results, it has been reported that mitochondria are a major source of LPS-stimulated ROS generation in microglia and that regulation of ROS production modulates, in turn, the production of proinflammatory mediators by preventing activation of the MAPK pathway induced by LPS and activation of NF-*κ*B in microglia [9].

Lu et al. [10] identified the involvement of ROS in the macrophage's polarization in the *Helicobacter pylori* infection. The study was carried out *in vitro* by coculturing EAW-364.7 cells with *H. pylori* at various multiplicities of infection (MOIs). They evaluated the macrophage polarization in M1 and M2 phenotypes, ROS production, and hypoxia-inducible factor 1*α* (HIF-1*α*). Macrophages were also treated with the ROS inhibitor NAC or HIF-1*α* inhibitor YC-1. The authors concluded that *H. pylori* enhances the ROS production and HIF-1*α* expression in macrophages and the MOI of *H. pylori* affects macrophage polarization state. These results rely on the crosstalk between ROS and HIF-1*α* that regulates *H. pylori*-induced macrophage polarization via the Akt/mTOR pathway. This study increases the current knowledge about the mechanisms involved in the macrophage polarization and gets the basis to the development of drugs able to modulate this process in the view of treatments of diseases such as atherosclerosis, enteritis, nephritis, tumour disease, and disorders of the nervous and skeletal system [11].

M. A. Lillo et al. [12] described in their research article the involvement of the superoxide anion in the signalling pathway dependent on pannexin-1 (Panx-1) for the control of endothelial cell function and NO-dependent relaxation. The authors demonstrate that blockade of Panx-1 channels leads to activation of Na_v_ channels and parallel recruitment of Panx-1 in caveolae, in association with Cav-1. The depolarizing current is mediated by the Na_v_ channel and is coupled to the opening of Cav_3.2_ and the subsequent entry of Ca^2+^. The concomitant depolarization of endothelial cells and the increase in [Ca^2+^]*_i_* results in the further activation of NADPH oxidase/O_2_^·–^ signalling, which triggers the PI3K/Akt pathway and the consequent increase in NO-mediated vasodilation through the modulation of eNOS activity of enzyme phosphorylation.

The signalling regulation of the signalling pathway by ROS is also relevant for glucose homeostasis, and it has been reported that it is involved not only in alterations in insulin signalling and the onset of insulin resistance [13] but also in glucagon secretion and the onset of type II diabetes, as summarized by A. N. Onyango in his review article [14]. In normal pancreatic islets, when plasmatic glucose concentration exceeds 7 mM, glucagon secretion is suppressed by the paracrine action of insulin and somatostatin produced by beta and delta cells, respectively. This paracrine suppression is lost in diabetes because alpha cells are resistant to insulin and somatostatin. Chronic exposure of alpha cells to elevated glucose levels upregulates SGLT-1 expression and activates the signalling pathway involving PI3K-Akt, PKC-*δ*, Src, and ROS in the islets of diabetic subjects. Oxidative stress and mitochondrial abnormalities cause reduced ATP production in alpha cells. The author reported that in addition to glucose, hydrogen peroxide promotes glucagon secretion and, if in excess, can induce oxidative stress and reduce ATP which are relevant in glucagon dysregulation in diabetes.

An interesting review of E. Maldonado et al. [15] describes the molecular mechanisms and the signalling pathways through which ROS produced in *Trypanosoma cruzi* infection (Chagas disease) induces beneficial effects on the pathogen and harmful effects on the host. The authors hypothesize that at the beginning of the infection, ROS produced by macrophages in response to *T. cruzi* infection can activate the MAPK transduction pathway in the pathogen that allows its growth and proliferation. In the later stages of infection, mitochondrial ROS are produced by infected cardiomyocytes that contribute to the oxidative damage that persists at the chronic phases of the disease. Oxidative damage leads to functional impairment of the heart. In this way, ROS trigger the growth and proliferation of the parasite and produce long persisting damage to the cardiomyocytes even in the absence of the parasite.

Involvement of ROS in signalling pathways is not only harmful to cells. ROS also play an important role in many homeostatic processes involving metabolism, immunity, growth, and differentiation; they are essential for proper cell development and proliferation, can have mitogenic effects, can mimic and amplify action of growth factors, and can induce the activation of antioxidant systems in response to environmental stimuli. The biological specificity of the actions is obtained through the quantity, duration of production, and location of ROS. This issue is examined in an interesting way by S. Di Meo and P. Venditti [16]. They point out that free radicals play a dual role in living systems: they are toxic by-products of aerobic metabolism, because they cause oxidative damage and tissue dysfunction, and they act as molecular signals by activating beneficial stress responses at low concentrations. From this point of view, the use of antioxidant molecules is generally considered useful to counteract the harmful effects of free radicals, but it is sometimes harmful as it can block adaptive responses induced by low radical levels [17–19].

In our opinion, the articles included in this special issue represent an important contribution to the knowledge concerning the cellular role of ROS, particularly referring to the involvement of these species in the regulation of signalling pathways.

*Sergio Di Meo* *Paola Venditti* *Victor M. Victor* *Gaetana Napolitano*
